# Generation of Fluorescent Bacteria with the Genes Coding for Lumazine Protein and Riboflavin Biosynthesis

**DOI:** 10.3390/s21134506

**Published:** 2021-06-30

**Authors:** Sunjoo Lim, Eugeney Oh, Miae Choi, Euiho Lee, Chan-Yong Lee

**Affiliations:** Department of Biochemistry, Chungnam National University, Daejeon 34134, Korea; sunjoo022@gmail.com (S.L.); komzzi@o.cnu.ac.kr (E.O.); miaechoi417@g.skku.edu (M.C.); ehlee@interejo.com (E.L.)

**Keywords:** bioluminescence, fluorescence, lumazine protein, *Photobacterium*

## Abstract

Lumazine protein is a member of the riboflavin synthase superfamily and the intense fluorescence is caused by non-covalently bound to 6,7-dimethyl 8-ribityllumazine. The pRFN4 plasmid, which contains the riboflavin synthesis genes from *Bacillus subtilis*, was originally designed for overproduction of the fluorescent ligand of 6,7-dimethyl 8-ribityllumazine. To provide the basis for a biosensor based on the *lux* gene from bioluminescent bacteria of *Photobacterium leiognathi*, the gene coding for N-terminal domain half of the lumazine protein extending to amino acid 112 (N-LumP) and the gene for whole lumazine protein (W-LumP) from *P. leiognathi* were introduced by polymerase chain reaction (PCR) and ligated into pRFN4 vector, to construct the recombinant plasmids of N-*lum*P-pRFN4 and W-*lum*P-pRFN4 as well as their modified plasmids by insertion of the *lux* promoter. The expression of the genes in the recombinant plasmids was checked in various *Escherichia coli* strains, and the fluorescence intensity in *Escherichia coli* 43R can even be observed in a single cell. These results concerning the co-expression of the genes coding for lumazine protein and for riboflavin synthesis raise the possibility to generate fluorescent bacteria which can be used in the field of bio-imaging.

## 1. Introduction

Bioluminescence and chemiluminescence refer to the process of visible light emission that occurs via exergonic chemical reactions, whereas fluorescence and phosphorescence involve remission of light from the singlet and triplet excited states, respectively [1]. Bioluminescence has long been used to image biological process in vivo. It can be detected in tissue and living organisms, providing sensitive and invasive data regarding physiological conditions [2].

**Figure 1:**
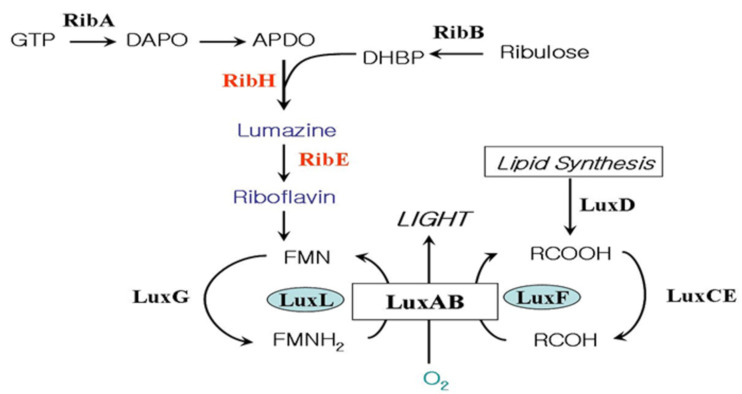
The genes involved in bacterial bioluminescence reaction of *Photobacterium* species. The functions of the gene products are as follow: luciferase (LuxAB), fatty acid reductase complex (LuxCDE), non-fluorescent flavoprotein (LuxF), flavin reductase (LuxG), lumazine protein (LuxL), GTP cyclohydrolase Ⅱ (RibA), dihydroxy-butanone 4-phosphate synthase (RibB), lumazine synthase (RibH), and riboflavin synthase (RibE).

Bacterial bioluminescence reaction involves the oxidation of long chain fatty aldehyde and reduced flavin mononucleotide (FMNH_2_) with the emission of greenish-blue light [3] (Figure 1).

FMNH_2_ + RCHO + O_2_ → FMN + H_2_O + RCOOH + light

**Figure 2:**
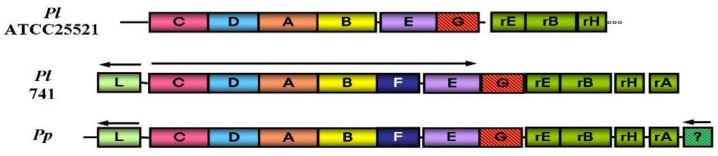
The gene organization of *lux* operon region in bioluminescence bacteria of *Photobacterium* species. *Photobacterium leiognathi ATCC 25,521* (Pl), *Photobacterium leiognathi* 741 (Pl), *Photobacterium phosphoreum* NCMB 844 (Pp). Arrow indicates the direction of transcription. ‘r’ represent for riboflavin and the functions of genes are shown in Figure 1.

The genes coding for the enzymes and proteins responsible for the light- emitting reaction in the bioluminescent bacteria exist in a cluster forming an operon [4]. The genes related to riboflavin (vitamin B_2_) biosynthesis on the downstream of the *lux* operon of *Photobacterium* species were reported [5,6,7] (Figure 2). The biosynthesis of riboflavin is essential in the bioluminescent bacteria, as it is a precursor of FMNH_2_ (riboflavin 5′-phosphate), which is the substrate of the bioluminescence reaction as shown in Figure 1. The detection of the riboflavin genes just downstream of *lux*G is particularly relevant to luminescence in *Photobacterium* species as this genus produces the highest level of light intensities of any luminescent bacteria, with luciferase levels reaching up to 20% of the soluble proteins [8].

The fact that the first gene (*rib*E) next to *lux*G codes for riboflavin synthase in *Photobacterium* species is interested in the aspect of molecular genetics. This protein is thought to be created through gene duplication, as it shows about 30% amino acid identity with riboflavin synthase and is under the superfamily of riboflavin synthase [6,9]. The lumazine protein, found in most *Photobacterium* species in 1970 [10], was the first antenna protein that shortens the wavelength and amplifies the maximum bioluminescence intensity in *Photobacterium* species [11]. The lumazine protein found in *Photobacterium* species are a paralog of riboflavin synthase which are devoid of enzymatic activity but bind the riboflavin synthase substrate, 6,7-dimethyl 8-ribityllumazine with high affinity, and serve as an optical transponder for bioluminescence emission [11,12].

**Figure 3:**
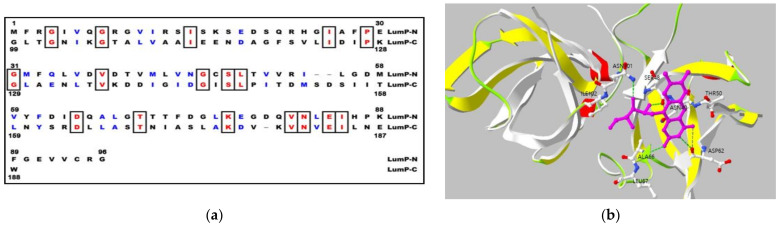
(**a**) Internal amino acid sequences between N-terminal domain half (LumP-N) and C-terminal domain half (LumP-C) of lumazine protein from *P. leiognathi* 741. Identical amino acids are marked in box and similar amino acids shown in blue letters; (**b**) Model of binding site topology of lumazine protein.

They show differences in the quaternary structure of protein, the lumazine protein has a monomeric structure whereas the riboflavin synthase forms trimeric structure [6,13]. The peculiar characteristics of lumazine protein is the intramolecular sequence similarity between N-terminal and C-terminal domain half (Figure 3a), and it was reported that the protein binds to one molecule of lumazine at N-terminal domain half [14,15] (Figure 3b). 

**Figure 4:**
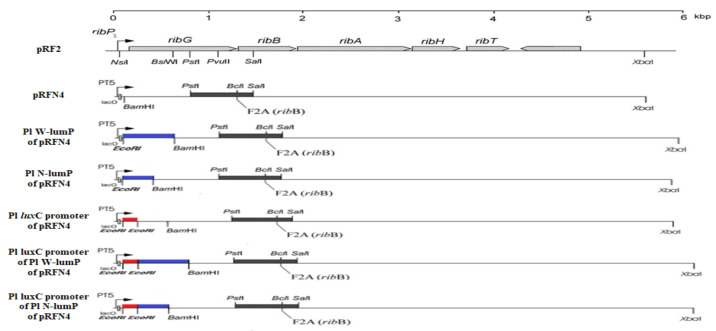
The riboflavin genes from *Bacillus subtilis* in the plasmids and the gene map of recombinant plasmids with inserting *Photobacterium leiognathi lux* genes. Cloning plasmid of pRF2, contains the entire riboflavin operon of *B. subtilis* inserted into the pNCO113 plasmid reprinted from reference [16], was designed for efficient expression in *E. coli*. The nomenclature of the gene coding for riboflavin synthase from *B. subtilis* (*rib*B) differs from that used for that in *Photobacterium* species (*rib*E). Therefore, *rib*B from *B. subtilis* and *rib*E from *Photobacterium* species genes have the same function of coding for riboflavin synthase. The lists of recombinant plasmids of pRFN4 using this study; pRFN4, Pl W-*lum*P of pRFN4, Pl N-*lum*P of pRFN4, Pl *lux*C promoter of pRFN4, Pl *lux*C promoter of Pl W-*lum*P of pRFN4, Pl *lux*C promoter of Pl N-*lum*P of pRFN4 plasmids.

In this research, we inserted a gene coding for the lumazine protein into the pRFN4 plasmid, which contains the riboflavin biosynthesis genes of *Bacillus subtilis,* in order to increase the fluorescence intensity since the lumazine chromophore binds to the lumazine protein with a high affinity. The pRFN4 recombinant plasmid containing whole riboflavin operon of *Bacillus subtilis* were generated to produce 6,7-dimethyl 8-ribityllumazine by the missense mutation of F2A in *rib*E gene for riboflavin synthase [16] (Figure 4).

Internal sequence similarity (Figure 3a) as well as the comparison with the paralogous of riboflavin synthase (RS), whose three-dimensional structure of N-terminal domain half has been determined by NMR and X-ray crystallization [17,18] (Figure 3b), suggest that lumazine protein folds into two domains with closely similar folding topology. Therefore, a truncated gene specifying the N-terminal domain half of the lumazine protein (N-LumP) was constructed and the cognate protein was expressed. Using the ligand of 6,7-dimethyl-8-ribityllumazine, the fluorescence characteristics of N-LumP was investigated and reveal that the N-terminal domain half itself can bind lumazine and emit fluorescence [19]. In the paper, the gene coding for half of the N-terminal domain of lumazine protein (N-LumP), as the minimal version of fluorescent lumazine protein, as well as the whole gene coding for the whole lumazine protein (W-LumP) were amplified by polymerase chain reaction (PCR) and inserted to pRFN4 to generate recombinant plasmids for producing of fluorescent bacteria (Figure 4).

In addition, we also inserted the DNA of the *lux* promoter region from *P. leiognathi* into the above recombinant plasmids to increase the expression of the lumazine protein gene. The *lux* promoter DNA from bioluminescent bacteria of *Photobacterium* species was amplified by PCR and ligated into pRFN4, Pl W-*lum*P of pRFN4, and Pl N-*lum*P of pRFN4 plasmids with the gene coding for whole lumazine protein (W-*lum*P) or N-terminal domain half of lumazine protein (N-*lum*P) from *P. leiognathi*. Therefore, the recombinant plasmids were constructed into Pl *lux*C promoter of pRFN4, Pl *lux*C promoter of Pl W-*lum*P of pRFN4, Pl *lux*C promoter of Pl N-*lum*P of pRFN4, respectively (Figure 4). We transformed into the several *E. coli* strains with the pRFN4 plasmid and the various recombined plasmids. Then, we conducted a spectroscopic study on the transformed *E. coli*, evaluating the fluorescence intensity and examining the fluorescence of a single cell by confocal microscopy.

## 2. Materials and Methods

### 2.1. Cloning Plasmids 

For the vectors, we used the PlXba.pT7-3 plasmid containing a gene of *Photobacterium leiognathi* (ATCC 25521) [20] and the pRFN4 plasmid containing a riboflavin synthesis genes of *B. subtilis* [16]. To amplify *lum*P gene, the DNA was amplified by PCR using plasmid pPHL36 [6] as template.

### 2.2. Strains and Cell Culture

For the cloning strain, we used *E. coli* XL-1 Blue (Table 1). Transformed *E. coli XL-1* Blue was incubated in Luria Bertani (LB) medium containing 100 μg/mL of ampicillin at 37 °C. We also used *E. coli* 43R for measuring fluorescence and imaging (Table 1).

### 2.3. Restriction Enzymes and Chemicals

*Eco*RI and *Bam*HI were purchased from New England Biolabs, PCR premix from Genet-Bio, and PCR primers from Bioneer. To amplify the gene coding for amino-terminal domain of lumazine protein, PCR was performed with Taq polymerase (New England Biolabs).

### 2.4. Generation of Recombinant Plasmids 

The PCR conditions were in five steps: pre-denaturation 95 °C 5 min, denaturation 9 °C 30 s, annealing 53 °C 30 s, extension 72 °C 80 sec, post-extension 72 °C 7 min. From the steps, denaturation, annealing, and extension were performed in 25 cycles. After the PCR and through the gel extraction, DNA and pRFN4 vector were cleaved with *Eco*RI and *Bam*HI restriction enzymes (Figure 4). After the restriction, ligation and transformation were performed with *E. coli* XL-1 Blue competent cell.

The genes coding for the N-terminal lumazine protein (N-LumP) extending the gene coding for the 112th position of amino acids in lumazine protein and for whole lumazine protein (W-LumP) from *P. leiognathi* 741 (Figure 3) were amplified by PCR, digested with *Eco*RI and *Bam*H1, and ligated into pRFN4 (Figure 4) to generate Pl N-*lum*P in pRFN4 and Pl W-*lum*P in pRFN4 recombinant plasmids. The forward primers for PCR were 5’ GGAGACCACAACGAATTCCCTCTAG3’ (Pl-*lum*P forward), 5’CAGAAAACCTGGGATCCTTCTATTCTATTGC3’ (Pl-N-*lum*P reverse), 5′GCTTGGGCTGGATCCGTTAATCACTAC3′(Pl-W-*lum*P-reverse). The restriction sites are underlined. The pPHl36 plasmid [6] containing the gene for lumazine protein from *P. leiognathi* was used as a template for PCR. 

To amplify the DNA of the *lux*C promoter region from *P. leiognathi*, we used the forward primer 5′-CATGAAAAATGAATTCTAAAAAAAT CAG- 3′ (Pl *lux*C promoter forward) and the reverse primer 5′ -CTTAATCATGAATTCTCCTTTG GTA- 3′ (Pl *lux*C promoter reverse). The site of restriction enzyme *EcoR*I are marked by underline in the DNA sequence of the primers. The 201 bp DNA was amplified by polymerase chain reaction (PCR) using the PlXba.pT7-3 plasmid [6] as the template. 

After these PCR processes, the amplified DNA was purified by agarose-gel electrophoresis, then the purified product digested with *Eco*RI, was inserted into the recombinant plasmids of Pl N-*lum*P of pRFN4 and of Pl W*-lum*P of pRFN4 cut by the same restriction enzyme *Eco*RI by ligation to generate additional recombinant plasmid of pRFN4, generating Pl *lux*C promoter of pRFN4, Pl *lux*C promoter of Pl W-*lum*P of pRFN4, Pl *lux*C promoter of Pl N-*lum*P of pRFN4, respectively (Figure 4). The various recombinant plasmids of pRFN4 DNA were transferred to the competent cells of *E. coli* XL-1. We incubated the colony and extracted a small amount of DNA. Finally, we confirmed the insertion of the right DNA sequence by the automated di-deoxynucleotide DNA sequencing analysis.

### 2.5. Fluorescence Measurement of Transformed E. coli

As a representative strain of transformation of recombinant DNA, the recombinant plasmids derived from pRFN4 were transferred into *E. coli XL-1 Blue*. In addition, the plasmids were also transformed into *E. coli* 43R which is known to the strain of overproduction of bioluminescence genes (Table 1). Then, to measure fluorescence, they were incubated in the conditions described as following. *E. coli* XL-1 Blue and *E. coli* 43R were incubated in LB medium without ampicillin, whereas *E. coli* cells harboring recombinant plasmids pRFN4, Pl-N-*lum*P in pRFN4, Pl-W-*lum*P pRFN4, as well as the recombinant plasmids of containing the promoter (Pl *lux*C promoter of pRFN4, Pl *lux*C promoter of Pl N-*lum*P of pRFN4, and Pl *lux*C promoter of Pl W-*lum*P of pRFN4) were grown LB media at 30 °C supplemented with 100 μg/mL ampicillin.

The cells were inoculated onto solid medium and after 24 h at 30 °C. A single bright colony was sterilely selected, inoculated in 100 mL liquid medium, and grown with shaking at 30 °C in a dark room to an absorbance of 2.5 at 600 nm. The constant amount growing cells (A_600_ × volume (ml) = 30) were harvested by centrifugation. The cell pellet was resuspended by buffer A (Tris-HCl 50 mM, EDTA 0.5 mM, DTT 0.5 mM, pH 7.2), sonicated (20 s, three times) and centrifuged to remove cellular debris. 

### 2.6. Fluorescence Spectroscopic Analysis

Using an LS 45 fluorescence spectrometer (PerkinElmer), we scanned the emission spectrum of each supernatant at the fixed excitation wavelengths of 410 nm and 450 nm, respectively. The molar absorptivity of 6,7-dimethyl-8-ribityllumazine and riboflavin are 10,300/M·cm and 13,153/M·cm, respectively [16,19].

### 2.7. Imaging of Fluorescent E. coli under Confocal Microscopy

The *E. coli* cells transformed with recombinant plasmids were incubated on poly-L-lysine coated slide glasses for 1 h and mounted sample using Antifade Mounting Medium with DAPI and FITC (Thermo Fisher Scientific). We examined the single cell image of the fluorescent *E. coli* with a Zeiss LSM 880 confocal laser-scanning microscope (Carl Zeiss, Oberkochen, Germany) and Zen^®^ Blue edition software (Zeiss).

## 3. Results

In addition to the amino acids such as Ser 48, Thr 50, and Ala 66 at the binding sites in N-terminal half of lumazine protein, several studies have shown that Asn 101 and Ile 102 located beyond N-terminal region are involved in binding of the lumazine ligand [14] (Figure 3a,b). Therefore, the genes coding for N-terminal domain half of lumazine protein extending to the amino acid 112 (N-LumP) and for the whole lumazine protein (W-LumP) from *P. leiognathi* were synthesized by PCR, ligated into pRFN4 vector to construct the recombinant plasmids of N-*lum*P-pRFN4 and W-*lum*P-pRFN4. We also conducted the PCR process inserting the *lux*C promoter domain, whose template is the PlXba.pT7-3 plasmid containing the *lux* genes *P. leiognathi* [20]. From these experiments, we generated that the recombinant plasmids, such as the Pl *lux*C promoter of pRFN4, the Pl *lux*C promoter of Pl W-*lum*P of pRFN4, and the Pl *lux*C promoter of Pl N-*lum*P of pRFN4, had sizes of around 9.0 kbp, 9.5 kbp, and 9.3 kbp, respectively (Figure 4).

**Figure 5:**
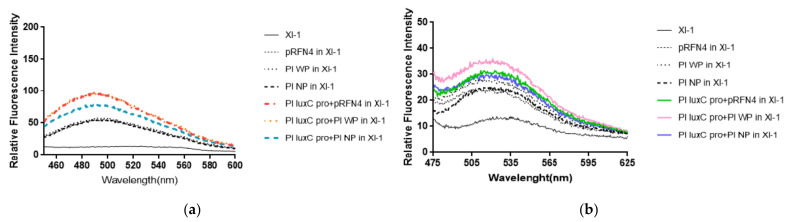
The fluorescence properties of *E. coli* XL-1 Blue transformed with recombinant plasmids of pRFN4 comtainin the *lux* gene from *P. leiognathi*. (**a**) Emission spectrum by lumazine in fixation at excitation wavelength of 410 nm. (**b**) Emission spectrum by riboflavin excited at excitation wavelength of 450 nm. NP and WP denote N-*lum*P and W-*lum*P, respectively.

Fluorescent intensities from *E. coli* cells harboring the recombinant plasmid containing the genes coding for the N-terminal domain half (N-LumP) and the whole lumazine protein (W-LumP) from *P. leiognathi* were tested. The fluorescence intensities of supernatants of the cells in buffer A was measured with spectrofluorimeter. It was observed that the supernatant before sonication also show fluorescent, indicating that the fluorophore was present in the liquid media. Fluorescence was detected by checking for the presence of 6,7-dimethyl-8-ribityllumazine (excitation at 410 nm, molar absorptivity 10,300/M·cm) and of riboflavin (excitation at 450 nm, molar absorptivity 13,153/M·cm). We fixed the excitation wavelength of lumazine at 410 nm and measured the emission spectrum. The fluorescence intensity of *E. coli* XL-1 Blue that was transformed with Pl W-*lum*P of pRFN4 was about 1.2∼2 times stronger than that of *E. coli* XL-1 Blue that was transformed with pRFN4 plasmid. The fluorescence intensity of *E. coli* XL-1 Blue that was transformed with pRFN4 plasmid containing the DNA of the *lux*C promoter region of *P. leiognathi* ATCC25521 was about 1.5∼2 times stronger than that of *E. coli* XL-1 Blue that was transformed with pRFN4 plasmid. Among all transformed *E. coli*, the fluorescence intensity was the highest in the *E. coli* XL-1 Blue that was transformed with the Pl W-*lum*P of pRFN4 plasmid containing the DNA of the *lux*C promoter region (Figure 5a).

Compared to the *E. coli* XL-1 Blue that was transformed with pRFN4 plasmid, the fluorescence intensity was stronger in the *E. coli* XL-1 Blue that was transformed with Pl W-*lum*P of pRFN4. This result is due to the non-covalent bonding between the lumazine protein and its chromophore lumazine. In addition, the fluorescence intensity can be stimulated due to the increase of expression of the genes coding for the lumazine protein by the insertion of DNA containing the region of the *lux* promoter region from *P. leiognathi*.

Based on a previous study that reported that riboflavin competes with lumazine to bind with the lumazine protein [13], we fixed the excitation wavelength of riboflavin at 450 nm and examined the emission spectrum. As you can see in Figure 5a, we found that the emission spectrum of riboflavin excited at 450 nm was similar to that of lumazine shown in Figure 5b. Compared to the *E. coli* XL-1 Blue that was transformed with pRFN4 plasmid, the fluorescence intensities from riboflavin was stronger in the *E. coli* XL-1 Blue that was transformed with Pl *lux*C promoter of Pl W-*lum*P of pRFN4 or Pl *lux*C promoter of Pl N-*lum*P of pRFN4.

**Figure 6:**
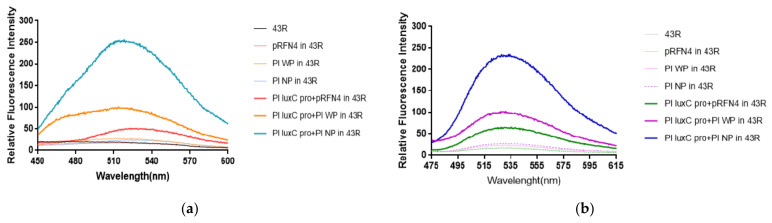
The fluorescence properties of *E. coli* 43R transformed with recombinant plasmids of pRFN4 containing the *lux* from *P. leiognathi* (**a**) Emission spectrum by lumazine excited at excitation wavelength of 410 nm. (**b**) Emission spectrum by riboflavin excited at wavelength of 450 nm. NP and WP denote N-*lum*P and W-*lum*P, respectively.

The author’s previous studies [20,21] show that luminescence intensity was significantly enhanced over 1000 times in *E. coli* 43R when transformed with the PlXba.pT7-3 plasmid among different *E. coli* strains. According to the result, we expected a larger increase of fluorescence intensity in *E. coli* 43R by the transformation of the pRFN4 recombinant plasmids compared to the transformation in *E. coli* XL-1, whereby *E. coli* transformed with pRFN4 plasmids show high fluorescence intensity (Figure 6a,b).

We examined the emission spectrum in the transformed *E. coli* 43R with the excitation wavelength fixed at 410 nm as we did in *E. coli* XL-1. As a result, the *E. coli* 43R transformed with Pl N-*lum*P of the pRFN4 plasmid showed about 1.5∼2 times stronger fluorescence intensity than the *E. coli* 43R transformed with the pRFN4 plasmid. Furthermore, the fluorescence intensity of the *E. coli* 43R transformed with the Pl *lux*C promoter of Pl N-*lum*P of pRFN4 was about 10 times stronger than that of the *E. coli* 43R transformed with the pRFN4 plasmid, and it was the strongest of all the transformed *E. coli* 43R (Figure 6a).

Accordingly, we suggest that this result is due to the increase of the binding of the lumazine ligand with the lumazine protein, as the *lux* promoter from *P. leiognathi* stimulates the expression of the gene coding the N-LumP. In addition, we fixed the excitation wavelength to that of riboflavin at 450 nm and examined the emission spectrum. In line with the emission spectrum of the excitation wavelength fixed to that of lumazine at 410 nm, the intensity of fluorescence was the strongest in the *E. coli* 43R transformed with the Pl *lux*C promoter of Pl N-*lum*P of the pRFN4 plasmid when the excitation wavelength was fixed at 450 nm (Figure 6b).

**Figure 7:**
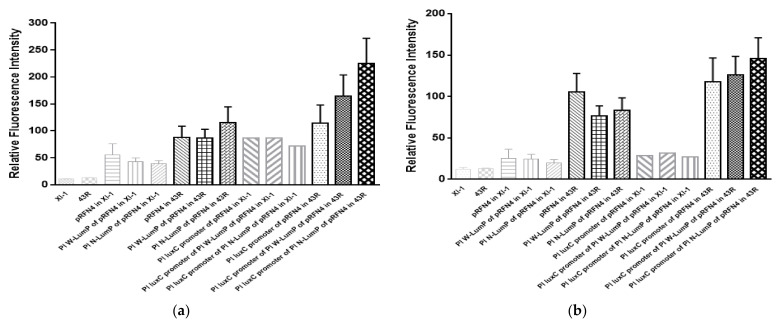
(**a**) Analysis of average fluorescence emission intensity from the different transformants. The x axis of bar graph means relative fluorescence intensity by lumazine in fixation at excitation wavelength of 410 nm from the *E. coli* transformed with recombinant plasmids from *P. leiognathi lux* gene. (**b**) Average emission value by riboflavin were obtained in fixation at excitation wavelength of 450 nm. The x axis of bar graph means relative fluorescence intensity from the *E. coli* transformed with recombinant plasmids from *P. leiognathi lux* gene.

Taken together, we analyzed the average value of the emission spectrum of lumazine and riboflavin (Figure 7a,b). The increase of the fluorescence intensity at 410 nm from the cell extract of *E. coli* XL-1 Blue to *E. coli* 43R was gradually increased by about 2∼6 times when transformed with the same plasmid (Figure 7a). Moreover, in the analysis of the mean value of the emission spectrum of riboflavin excited at 450 nm, the increase of the fluorescence intensity from *E. coli* XL-1 Blue to *E. coli* 43R was sharp and varied by about 5∼6 times when transformed with the same plasmid (Figure 7b). Therefore, the highest fluorescence intensity of the cell containing the recombinant plasmid of Pl *lux*C promoter of Pl N-*lum*P of pRFN4 has a value 250 times higher compared to the intensity from *E. coli* XL or *E. coli* 43R itself.

**Figure 8:**
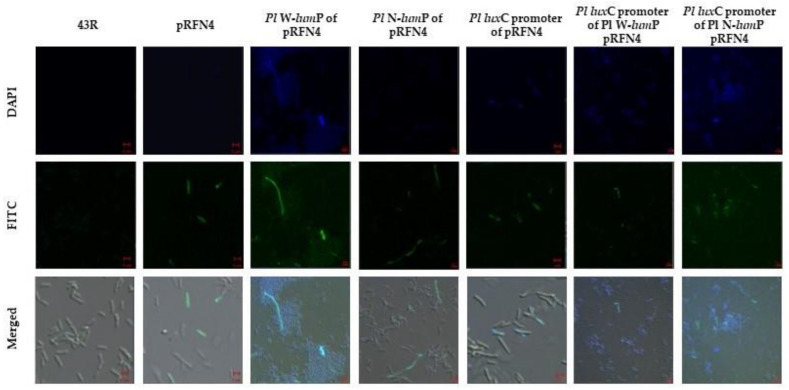
The microscopic images of *E. coli* 43R containing the recombinant plasmids of pRFN4 inserted the *lux* genes from *P. leiognathi*.

Finally, we checked the imaging of fluorescent *E. coli* with confocal microscopy. The *E. coli* 43R that was transformed using the recombination plasmid with the *lux* gene of *Photobacterium* species was incubated in a small amount of LB medium. We examined the single cell image of fluorescent *E. coli* using DAPI (excitation 405 nm) and FITC (excitation 458 nm) filter sets in the super-resolution confocal laser scanning microscope LSM880. The *E. coli* 43R that was not transformed did not show any fluorescence. On the other hand, some of the *E. coli* 43R that was transformed with the recombinant plasmids of pRFN4 showed fluorescence (Figure 8). We observed fluorescence in the single cell of *E. coli* 43R transformed with the recombinant plasmids of pRFN4 that was inserted with the genes for W-LumP and N-LumP. 

The fluorescence was stronger and observed in more cells compared to the *E. coli* 43R transformed with only the pRFN4 plasmid. Furthermore, in the *E. coli* 43R transformed with the recombinant plasmid that was additionally inserted with the DNA of the *P. leiognathi lux*C promoter domain, the fluorescence was more intense and most of the cells expressed the fluorescence as shown in the image (Figure 8). The fluorescence intensities and frequencies in a single cell of *E. coli* transformed with the recombinant plasmids containing the gene coding for the lumazine protein from *P. leiognathi* correlated with the result with the fluorescence intensity shown in Figure 6.

## 4. Discussion

Bioluminescence imaging of a single cell is often complicated by the requirements of exogenous luciferin that can be poorly cell permeable or produce a high background signal [3]. Gregor et al. have engineered an improve operon *ilux*, which enable long term visualization of single bacterial cells while simultaneously providing information about cellular viability [22]. Bacterial bioluminescent system and light emission is being applied sensitive and safe assay not only for prokaryote gene expression but single mammalian cells [23,24]. Gregor et al. reported high luminescence levels that support the autonomous bioluminescence microscopy of mammalian cells [23].

We inserted the genes for the lumazine protein and the DNA of the *lux* promoter region of *Photobacterium* species into the pRFN4 plasmid that overproduces the chromophore lumazine, generating to various recombinant plasmids such as Pl W-*lum*P of pRFN4 and Pl N-*lum*P of pRFN4 plasmids that contain the lumazine protein gene of *P. leiognathi*. Then, these recombinant plasmids were transformed into the *E. coli*. We evaluated the fluorescence intensity using a fluorescence spectrometer and performed an imaging study of a single cell through confocal microscopy in each type of transformed *E. coli*. The *E. coli* cells that were transformed with the pRFN4 plasmids more clearly expressed the fluorescence by the overproduced lumazine ligand compared to the *E. coli* that was not transformed. The fluorescence was significantly stronger when *E. coli* was transformed with the recombinant plasmid that was also inserted with the lumazine protein gene of *P. leiognathi* with the *lux*C promoter. This gives spectroscopic evidence that the fluorescence intensity is increased when the lumazine protein is expressed along with the chromophore lumazine or riboflavin.

The recombinant plasmid DNA was transformed into *E. coli* strain 43R and the fluorescence intensity of the bacteria was measured. As a result, the recombinant plasmid in which the gene coding for the lumazine protein and the DNA containing *lux* promoter, the expression of this gene was so high to show fluorescence in single colony of *E. coli*. The increasing variety of fluorescent proteins and FRET (fluorescence resonance energy transfer) has significantly impacted many molecular and cellular investigations [25]. Therefore, the fluorescent system using bacterial bioluminescence genes can be utilized as a biosensor with high sensitivity and short analysis time.

In this study, we constructed series of pRFN4 recombinant plasmid derivatives, including Pl W-*lum*P of pRFN4, and Pl N-*lum*P of pRFN4 plasmids, with the gene coding for whole lumazine protein (W-LumP) or N-terminal domain half of lumazine protein (N-LumP) from *P. leiognathi* as well as Pl *lux*C promoter of pRFN4, Pl *lux*C promoter of Pl W-*lum*P of pRFN4, and Pl *lux*C promoter of Pl N-*lum*P of pRFN4 inserted the *lux* promoter DNA from bioluminescent bacteria of *Photobacterium* species, respectively. By inserting of the *lux*C promoter region into the plasmids, the fluorescent ligand largely bound the lumazine protein because of the high expression of the gene for lumazine protein by *lux* promoter in the transformed the *E. coli*. The fluorescent intensity of these transformed the *Escherichia coli* were measured by fluorescence spectrometer, and a single cell of the transformed *E. coli* was observed the appearance of fluorescence by using confocal microscopy. Notably, the expression of recombinant plasmids in *E. coli* 43R was so strong as to allow observation of the fluorescence in a single cell, raising the possibility for the generation of fluorescent bacteria which can provide the basis for a microbial biosensor.

There has been drastic interest in fluorescence for cellular and molecular imaging [26,27], and fluorescence is now a dominant methodology applied to biotechnology, flow cytometry, DNA sequencing, and genetic analysis. Fluorescence imaging can provide the information on the localization and measurement of intracellular molecules [26]. Many genetically encoded proteins that possess fluorescence and FRET have been developed to better understand the spatiotemporal regulation of various cellular process [26,27]. Whole cell sensing systems based on bioluminescence have the benefit of detecting a variety of environmental pollutants [2,28]. Therefore, the results presented in this paper raise the possibility that the *Escherichia coli* 43R harboring the recombinant plasmid inserted the genes coding for lumazine protein and riboflavin biosynthesis can be used as fluorescent bacteria for application in the field of bio-imaging.

## Figures and Tables

**Table 1:** Bacterial strains and plasmids used in this study.

**Strains**	**Characteristics**	**Source**
*E. coli* XL-1 Blue	Cloning strain	Real Biotech corporation
*E. coli* 43R	Mutant of *E. coli* RR1 strain	C. Miyamoto et al., [20]
**Plasmids**	**Characteristics**	**Source**
pRFN4	Recombinant plasmid containing the riboflavin genes from *Bacillus subtilis*	Illarionov, B., et al., [16]
pPhl36	pT7-7 containing the gene for the wild type of lumazine protein from *Photobacterium leiognathi* 741	Illarinov, B., et al., [6]
PlXba in pT7	The recombinant pT7-3 plasmids containing the cloned *P.leiognathi* ATCC 25,521 *lu0078* DNA	Lee et al., [21]
